# Sodium valproate enhances efficacy of NKG2D CAR-T cells against glioblastoma

**DOI:** 10.3389/fimmu.2024.1519777

**Published:** 2025-01-14

**Authors:** Junchen Liu, Kun Dai, Muhammad Auwal Saliu, Mansur Dabai Salisu, Jiangyu Gan, Lukman Olalekan Afolabi, Dehong Yan, Guizhong Zhang, Maoxuan Liu, Xiaochun Wan

**Affiliations:** ^1^ Guangdong Immune Cell Therapy Engineering and Technology Research Center, Center for Protein and Cell-based Drugs, Institute of Biomedicine and Biotechnology, Shenzhen Institute of Advanced Technology, Chinese Academy of Sciences, Shenzhen, China; ^2^ University of Chinese Academy of Sciences, Beijing, China; ^3^ Inspection Department, Ji’an Central People’s Hospital, Ji’an, China; ^4^ Department of Pediatrics, Indiana University School of Medicine, South Bend, IN, United States

**Keywords:** CAR-T, glioblastoma, NKG2D, sodium valproate, combination therapy

## Abstract

Chimeric antigen receptor T-cell (CAR-T) therapies have shown promise in glioblastoma clinical studies, but responses remain inconsistent due to heterogeneous tumor antigen expression and immune evasion post-treatment. NKG2D CAR-T cells have demonstrated a favorable safety profile in patients with hematologic tumors, and showed robust antitumor efficacy in various xenograft models, including glioblastoma. However, malignant glioma cells evade immunological surveillance by reducing NKG2D ligands expression or cleavage. To enhance the effectiveness of NKG2D CAR-T therapy, we investigated the potential of combining NKG2D CAR-T with approved drugs that cross the blood-brain barrier and augment NKG2D ligands expression in glioma cells. We found that sodium valproate (VPA), an antiepileptic drug, significantly increased surface NKG2D ligands expression on glioblastoma cells at a sublethal concentration. VPA treatment enhanced the susceptibility of glioblastoma cells to NKG2D CAR-T mediated cytotoxicity in both 2D monolayer and 3D tumor spheroid models *in vitro*. Moreover, VPA-treated glioblastoma cells stimulated CAR-T cells to produce higher levels of inflammatory cytokines (IL-2, IFN-γ, and IL-6). Mechanistically, VPA upregulated NKG2D ligands expression via the PI3K/Akt signaling pathway. Additionally, VPA treatment augmented the antitumor activity of NKG2D CAR-T cells in a glioblastoma xenograft model *in vivo*. These preclinical results suggest that combining VPA with NKG2D CAR-T therapy represents a promising strategy for improving glioblastoma treatment, warranting further clinical investigation.

## 1 Introduction

Glioblastoma is the most common and lethal malignant primary brain tumor in adults. Glioblastoma treatment options currently include surgical resection, radiotherapy and chemotherapy. However, the 5-year survival rate of glioblastoma patients remains less than 10%, and the median survival is < 21 months (1, 2). Surprisingly, the overall survival rate of glioblastoma has not changed significantly over the last few decades, whereas survival rates for many other cancers have improved significantly due to advances in early detection, the introduction of precision medicine, and treatment advances (3). Therefore, there is an urgent need for the development of new glioblastoma treatment strategies.

CAR-T cells are T cells engineered to express a specific CAR (chimeric antigen receptor) that recognizes tumor-associated antigens (TAA) on the surface of tumor cells and kills them in an MHC-independent manner. CAR T-cell therapy has resulted in excellent clinical outcomes in hematologic cancers. So far, the FDA has approved six CAR-T products for the treatment of hematological malignancies (4). CAR-T cell therapy has also been studied in solid tumors, including glioblastoma. Several CARs have been described in recent clinical studies for glioblastoma, including EGFR variant III (EGFR VIII), IL13 receptor subunit alpha 2 (IL13Ra2) and HER2 (5). Although these CAR-T therapies showed promising results and represented a treatment option for glioblastoma, their antitumor activity was limited in early phase clinical testing, in part due to heterogeneous tumor antigen expression and escape following CAR-T cell treatment (6, 7). Therefore, it is critical to develop more effective CAR-T cell-based therapy for the treatment of glioblastoma.

NKG2D is a highly conserved activating receptor expressed on NK cells and CD8^+^ T cells, recognizing eight NKG2D ligands (NKG2DL), including MICA, MICB, and the UL16-binding proteins (ULBP1–6). NKG2DL expression has been reported in a wide range of solid tumors and hematologic malignancies, however, these ligands are generally absent in healthy tissues. NKG2D CAR-T targeting NKG2DL has demonstrated potent antitumor efficacy in a variety of xenograft tumor models (8). Besides, a phase I trial of autologous NKG2D CAR-T in patients with hematological malignancies revealed that NKG2D CAR-T cells have a good safety profile (9), indicating that NKG2DL is a potential target for CAR-T therapy. Most gliomas express MICA/B and ULBP proteins, and NKG2D CAR-T has recently been shown to be effective against glioblastoma (10). However, when compared to other types of cancer, glioblastoma exhibited moderate expression of NKG2D ligands (The Human Protein Atlas database: https://www.proteinatlas.org/). Furthermore, in some cases, malignant glioma cells evade immunological surveillance by reducing NKG2D ligand expression or cleavage, which may limit the use of NKG2D CAR-T for treating glioblastoma (11). As a result, combining NKG2D CAR-T with agents that boost NKG2DL expression in glioma cells is a promising strategy for improving glioblastoma treatment efficacy.

For more than five decades, sodium valproate (VPA) has been used as an antiepileptic drug. Recent studies have demonstrated that VPA can increase the surface expression of NKG2D in acute myeloid leukemia, renal carcinoma and ovarian cells (12–14). Given that VPA can cross the blood-brain barrier, we investigated the effect of VPA on the expression of surface NKG2D ligands in glioma cell lines and attempted to combine VPA and NKG2D CAR-T to treat glioblastoma in this study. We show that VPA can increase NKG2DL expression in glioblastoma cell lines and improve the killing capacity of NKG2D CAR-T cells. This research offers a novel and promising therapeutic strategy for glioblastoma.

## 2 Materials and methods

### 2.1 Cell culture

U87 cells were obtained from the American Type Culture Collection (ATCC, USA). U251 and A172 cells were kindly provided by the Chinese Academy of Sciences’ Cell Bank/Stem Cell Bank. Cancer cells were cultured in DMEM medium supplemented with 10% fetal bovine serum (Gibco, USA), 100 μg/ml streptomycin, and 100 U/ml penicillin (Hyclone, USA). All the cells were incubated at 37°C with 5% CO_2_.

### 2.2 Reagents and antibodies

Sodium valproate was obtained from Solarbio Life Science (China). DMEM media, Opti-MEM I Reduced Serum Medium, and Fetal Bovine Serum (FBS) were obtained from Gibco (USA). APC anti-human CD314 (NKG2D) antibody(Cat# 320807), APC anti-human MICA/B antibody (Cat# 320907), APC anti-human CD314 (NKG2D) antibody(Cat# 320807), APC mouse IgG2a isotype control antibody (Cat# 400219), APC Goat anti-mouse IgG antibody (Cat# 405308), APC anti-CD3 antibody (Cat# 300411) and PE anti IFN-γ (Cat# 502508) were obtained from BioLegend (USA). Anti-human ULBP1 antibody (Cat# MAB1380-SP), anti-human ULBP2/5/6 antibody (Cat# MAB1298), anti-human ULBP3 antibody (Cat# MAB1517), and mouse IgG2a isotype control (Cat# MAB003) were obtained from R&D Systems (USA). Anti-human ULBP4 antibody (Cat# sc-53133) was obtained from Santa Cruz Biotechnology (USA). Human Th1/Th2 Cytokine Cytometric Bead Array (CBA) Kit (Cat#550749), and Cytofix/Cytoperm fixation and permeabilization kit (Cat#554714) were obtained from BD biosciences (USA).

### 2.3 Plasmid construction and lentiviral package

The extracellular domain of human NKG2D was synthesized (Genewiz) and fused to a CAR backbone comprising a human CD8a hinge spacer and transmembrane domain, 4-1BB costimulatory domain, and CD3ζ. The entire encoding sequence of the CAR expression molecule was cloned into the lentiviral vector pWPXLd (Addgene). For the lentiviral package, the lentiviral plasmids were co-transfected into HEK293T cells with the packaging plasmids psPAX2 and pMD2.G (Addgene) at a ratio of 5:3:2. Lentivirus was harvested 48h after transfection.

### 2.4 Generation of CAR-T cells

The CAR-expressing lentiviral vectors were transduced into human primary T cells as previously described, with some modifications (15). T cells were isolated from peripheral blood mononuclear cells (PBMC) obtained from healthy donors and activated with Dynabeads™ CD3/CD28 (Thermo Fisher Scientific) at a 1:1 ratio and then cultured in Corning^®^ KBM 581 serum free medium containing IL-2 (50 U/mL, Novoprotein). After 24 h, activated T cells were infected with the lentiviral particles. Seven days after stimulation, the beads were removed. The percentage of NKG2D^+^ cells was used to calculate transduction efficiency using flow cytometry. All the studies involving human subjects were approved by the Institutional Review Board at Shenzhen Institute of Advanced Technology, Chinese Academy of Sciences (approved ID: SIAT-IRB-190715-H0363) with written informed consent obtained from participants and conducted in accordance with the international ethical guidelines for biomedical research involving human subjects.

### 2.5 Flow cytometry

Cells were harvested, washed twice with 1xPBS, and resuspended at a density of ~1×10^6^ cells/mL in cold PBS containing 0.5% BSA. Subsequently, labeled primary antibodies were added to the cell suspension according to the manufacturer’s instructions and incubated at 4°C in the dark for 0.5 h. All flow cytometry measurements were taken with the Beckman CytoFLEX flow cytometer system and analyzed with the FlowJo software.

### 2.6 Pretreatment of cancer cell lines with VPA

The cytotoxicity of VPA on glioblastoma cell lines U251, U87, and A172 was assessed using the Cell Counting Kit-8 (CCK-8) assay. Glioblastoma cells were seeded at a density of 5×10^4^ cells/mL in 96-well plates. Cells were incubated for 48 h with increasing concentrations of VPA after 24 h of attachment (0, 0.4, 0.8, 1.6, 3.2, 6.4 and 12.8 mM). Cell viability was determined using a CCK-8 kit (Shanghai Yeasen Biotech) as described by the manufacturer. For flow cytometric analysis, Glioblastoma cells were seeded in 12-well plates at a cell density of 1×10^5^ cells/mL. Cells were incubated with 3.2 mM VPA for 48 h after 24 h of attachment. NKG2D ligands were detected using antibody-based flow cytometry, as previously described. For the LY294002 and PD98059 experiments, the pathway inhibitors were combined with VPA and applied to the target cells.

### 2.7 Cytotoxicity assay

Cytotoxic activity of CAR-T cells *in vitro* was determined using the xCELLigence RTCA SP instrument (ACEA Biosciences) with minor modification (16). Target cells were seeded at a density of 5×10^3^ cells/well into the plate and cultured for ~24 h. Data recording was paused at the time of initiating treatments, and CAR-T or untransduced (UTD) cells were added into the target cells at an indicated effector: target (E: T) ratio in triplicate. The target cell-only control was included. The cytotoxic activity based on the viability of attached target cells was monitored for at least 24 h, as reflected by cell index (CI) values. CI was normalized at the end of the experiment to remove any well-well variation using the RTCA software Pro (version 2.3.0). Percentage cytotoxicity was calculated at the end of the experiment as:

% cytotoxicity = ((CI no effector - CI effector)/(CI no effector)) × 100.

In the experiments involving VPA treatment, 3.2 μM of VPA or PBS vehicle control was used to pretreat target tumor cells (U251, U81, and A172) for 48 h, and media was changed to complete media. NKG2D CAR-T cells were added directly to the pretreated cells at different E: T ratios.

In the 3D spheroid model, U251 cells (5×10^3^/well) were seeded in Corning^®^ spheroid 96-well microplates and incubated for 72 h under physiological conditions to form the required U251 spheroids. After 3.2 mM VPA treatment for 72 h, then NKG2D CAR-T cells were added at an E: T ratio of 2 with 0.5% propidium iodide solution (BioLegend). The red fluorescence intensity values in each well were collected and analyzed by Cytation 5 Cell Imaging Multi-Mode Reader (BioTek, USA) over 24 h co-culture.

### 2.8 Cytokine release assays

After co-culturing CAR-T cells and glioblastoma cell lines for 24 h, the culture supernatant was collected through centrifugation at 300 g for 5 minutes and transferred to new flat-bottom 96-well plates. The cytokine levels Interleukin-2/4/6/10, interferon-gamma (IFN-γ) and tumor necrosis factor-alpha (TNF-α) were quantified by Human Th1/Th2 Cytokine Cytometric Bead Array (CBA) Kit (BD Biosciences) according to the manufacturer’s instructions. Data were analyzed using FlowJo software.

### 2.9 Intracellular staining of IFN-γ

T cells (5 × 10^5^) were co-cultured with target cells treated with VPA or not at a ratio of 1:1 for 24 h followed by incubation with Brefeldin A (BD Biosciences) for 4 h. Cytofix/Cytoperm fixation and permeabilization kit (BD Biosciences) was used to permeabilize cell membrane and perform intracellular staining according to the manufacturer’s instruction. APC anti-CD3 antibody and PE anti-IFN-γ antibody were used for immunostaining. IFN-γ expression was detected via antibody-based flow cytometry as described previously.

### 2.10 Quantitative real-time RT-PCR

Total RNA was extracted from cells using RNAeasy™ Animal RNA Isolation Kit with Spin Column (Beyotime Biotechnology) and reverse transcribed into cDNA using the EasyScript™ First-Strand cDNA Synthesis SuperMix (Shanghai Yeasen Biotech). The expressions of MICA, MICB, ULBP1, ULBP2, ULBP3, ULBP4, ULBP5, ULBP6, and GAPDH were quantified using the quantitative SYBR Green PCR kit (Shanghai Yeasen Biotech) according to the manufacturer’s protocol. The primers used for qRT-PCR are shown in **Supplementary Table S1**.

### 2.11 *In vivo* glioblastoma xenograft mouse model

Mouse experiments were performed in accordance with relevant guidelines and regulations and were approved by the Institutional Animal Care and Use Committee at Shenzhen Institute of Advanced Technology, Chinese Academy of Sciences. Mice were maintained under specific-pathogen-free conditions with daily cycles of 12-h light–12-h darkness and health monitoring was carried out on a regular basis. Six-week-old female B-NDG mice used in this study were purchased from Biocytogen (Beijing, China). The mice were subcutaneously injected with 2 × 10^6^ U251 tumor cells. Tumor growth was monitored every 3 days. When the tumor volume reached around 100mm^3^ (calculated as volume = (width)^2^ x length/2), the mice were treated by VPA at a dosage of 100mg/kg, or a vehicle control intravenously every two days. After VPA was administered 4 times, 5.0×10^6^ NKG2D CAR-T or untransduced T (UTD) cells were administered intravenously to the mice. Throughout the experiment, tumor volume and body weight of mice were measured and monitored. The study was concluded on day 51, and all animals were euthanized. Tumor tissues and peripheral blood samples were collected from each mouse for further analysis.

### 2.12 Statistical analysis

The results are presented as the mean ± SD. Normality was assessed using the Shapiro-Wilk test, and homogeneity of variances was evaluated with the Levene test. For comparisons between two groups, a two-tailed t-test was applied when normality was not rejected, while one-way ANOVA with a Tukey’s multiple-comparisons test was used for comparisons for multiple groups. When normality was rejected, the Mann-Whitney test was used for two-group comparisons, and the Kruskal-Wallis test for multiple groups comparisons. Statistical significance was indicated by an asterisk (*) for *p* < 0.05 between the specified groups. Experimental sample numbers (*n*) are indicated in figure legends.

## 3 Results

### 3.1 NKG2D CAR-T cells efficiently lyse glioblastoma cells

**Figure 1:**
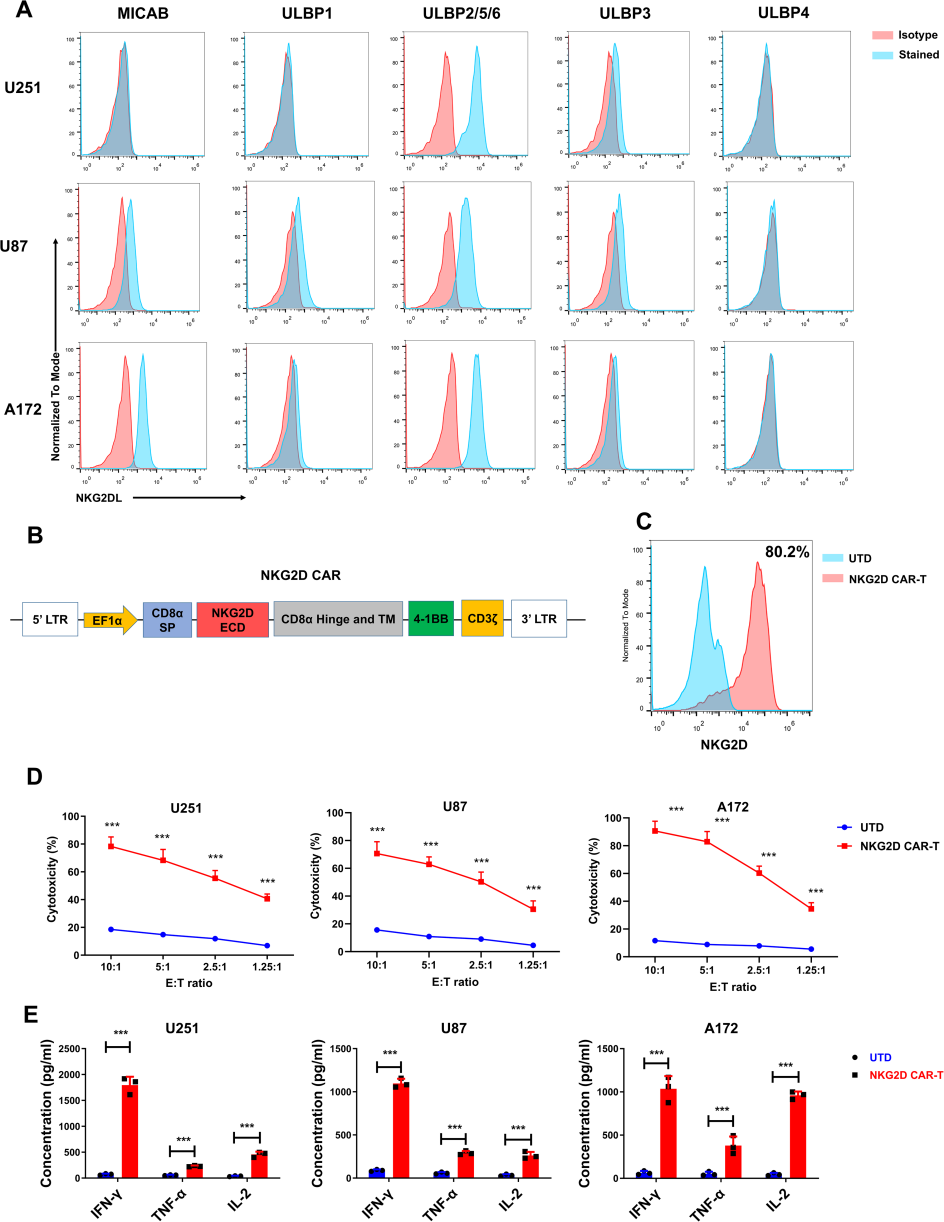
NKG2D ligands are highly expressed in glioblastoma cell lines, and NKG2D CAR-T cells efficiently lyse glioblastoma cells *in vitro*. **(A)** NKG2D ligands expression in U251, U87 and A172 cell lines was detected by flow cytometry. Data are representative of three independent experiments. **(B)** Schematic representation of lentiviral vector expressing NKG2D CAR. **(C)** NKG2D CAR expression on human T cells transduced with a lentivirus and analyzed using flow cytometry. Data are representative of three independent experiments. Percentage of positive cells is detailed in the histograms. **(D)** The cytotoxicity of untransduced cells (UTD) and NKG2D CAR-T cells against the indicated cell lines at different effector to target (E: T) ratios after 24 h co-culture. The results are presented as the mean ± SD (*n* = 3). **(E)** The levels of cytokines (IFN-γ, IL-2 and TNF-α) released by UTD control and NKG2D CAR-T cells were measured by cytometric bead array (CBA) assay after 24 h co-culture incubation at an E:T ratio of 1.25. The results are presented as the mean ± SD (*n* = 3). Statistical significance between the NKG2D CAR-T group and UTD control group was calculated using a two-tailed unpaired Student t test (***P ≤ 0.001).

Three representative glioblastoma cell lines (U251, U87, A172) were selected for assessing cell surface expression of NKG2DL (MICA/B, ULBP1, ULBP2/5/6, ULBP3, and ULBP4) by flow cytometry. As expected, all the tested glioblastoma cell lines expressed high levels of NKG2DL, particularly ULBP2/5/6 (**Figure 1A**). The expression of NKG2DLs in glioblastoma cells suggested that NKG2D CAR-T cells could be used as potential therapy for glioblastoma. Therefore, we constructed an NKG2D-CAR lentiviral vector with the NKG2D extracellular region, 4-1BB, and CD3 signaling domains (**Figure 1B**).

The NKG2D-CAR lentivirus was used to transduce T cells from healthy donors, and the expression of NKG2D CAR was assessed by flow cytometry 5 d post transduction. As shown in **Figure 1C**, a relatively high percentage of T cells (approximately 80%) were positive for NKG2D CAR expression. To assess the cytotoxicity of NKG2D CAR-T cells against glioblastoma cells, we incubated CAR-T and untransduced control T (UTD) cells with target cells (U251, U87, and A172) at various E: T ratios (10:1, 5:1, 2.5:1, and 1.25:1). When compared to UTD cells, NKG2D CAR-T cells lysed the glioblastoma cells more efficiently (**Figure 1D**).

Additionally, since cytokine secretion by CAR-T cells targeting cancer cells indicates T cell activation and specific cytotoxicity, the levels of the classic cytokines (IFN-γ, IL-2, and TNF-α) were measured when CAR-T cells were incubated with glioblastoma cells to assess the cytokine profile. In comparison to control cells, the concentrations of tested cytokines were significantly higher in the supernatant of the NKG2D CAR-T co-culture system (*P* < 0.001, **Figure 1E**).

These findings suggested that glioblastoma cell lines overexpress NKG2D ligands and NKG2D CAR-T can efficiently lyse glioblastoma cells. As a result, NKG2D CAR-T cells could be a promising therapeutic approach for patients with glioblastoma.

### 3.2 VPA treatment increases NKG2D ligands expression and enhances susceptibility of glioblastoma cells to NKG2D CAR-T mediated cytotoxicity

The safe and effective clearance of cancer cells by CAR T cells necessitates both selective and consistent cell surface expression of the target antigen. All components of an antitumor CAR T cell response can be affected by the density of antigen expression (17). We analyzed the expression of NKG2D ligands in different types of human cancers using The Human Protein Atlas Database (http://www.proteinatlas.org). Glioblastoma exhibited moderate expression of the NKG2D ligands when compared to other types of cancer. Furthermore, glioblastoma has been shown to evade NKG2D-mediated immune detection by shedding soluble NKG2D ligands from the cell surface (11). The level of expression of the target antigen on the surface of tumor cells has a direct impact on the therapeutic effect of CAR-T cells (18). As a result, we hypothesized that pharmacologically increasing NKG2D ligand expression would improve glioblastoma cell recognition and susceptibility to NKG2D CAR-mediated immune response.

**Figure 2:**
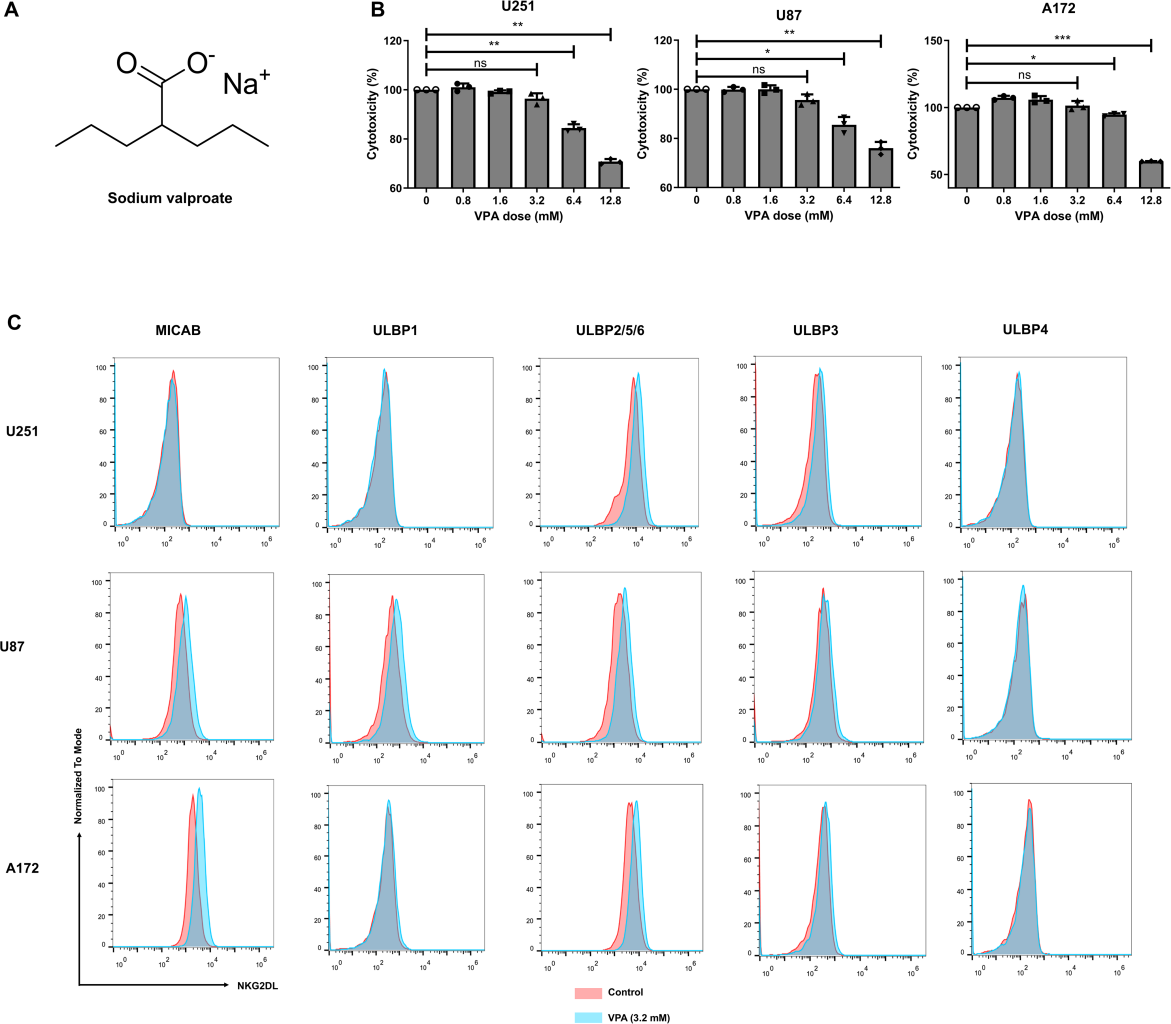
Sodium valproate (VPA) treatment increased NKG2D ligands expression of glioblastoma cell lines at subtoxic concentration. **(A)** Chemical structure of VPA. **(B)** Effect of VPA on glioblastoma cells viability. The viability of glioblastoma cancer cells was analyzed using Cell Counting Kit-8 (CCK-8) assay 48 h after VPA treatment (*n* = 6). Statistical significance was calculated using one-way ANOVA (*P ≤ 0.05, **P ≤ 0.01, ***P ≤ 0.001). **(C)** The surface expression of the respective NKG2D ligands in U251, U87 and A172 cell lines treated with 3.2 mM VPA or vehicle control for 48 h.

VPA (**Figure 2A**) is an antiepileptic drug that is a histone deacetylase (HDAC) inhibitor and has been shown to increase the surface expression of NKG2DL on several cancer cells (12–14). However, whether VPA increases the surface expression of NKG2D ligands on glioblastoma cancer cells has not been known. Considering the ability of VPA to cross the blood-brain barrier, we tested VPA’s ability to sensitize glioblastoma cancer cells to NKG2D CAR-T cytotoxicity.

To investigate this, we first looked for an *in vitro* VPA dose at which most glioblastoma cancer cells remained viable. The viability of the three glioblastoma cell lines was assessed after 48 h of treatment with increasing doses of VPA (0, 0.8, 1.6, 3.2, 6.4, or 12.8 mM). VPA at the concentration of 3.2 mM and lower had negligible cytotoxicity on these cell lines, with > 95% cancer cell viability (**Figure 2B**). We then used flow cytometry to examine the cell surface expression levels of NKG2DL on cancer cells after 48 h of exposure to 3.2 mM VPA (the subtoxic concentration). When compared to the respective untreated controls, all three glioblastoma cell lines treated by VPA showed an increase in NKG2DL expression (**Figure 2C**).

Specifically, all cell lines showed increased ULBP2/5/6 expression. MICA/B expression was also significantly increased on U87 and A172 cells, and ULBP3 expression was increased on U251 cells. On the other hand, VPA treatment had no effect on ULBP4 ligand expression in the glioblastoma cells tested.

**Figure 3:**
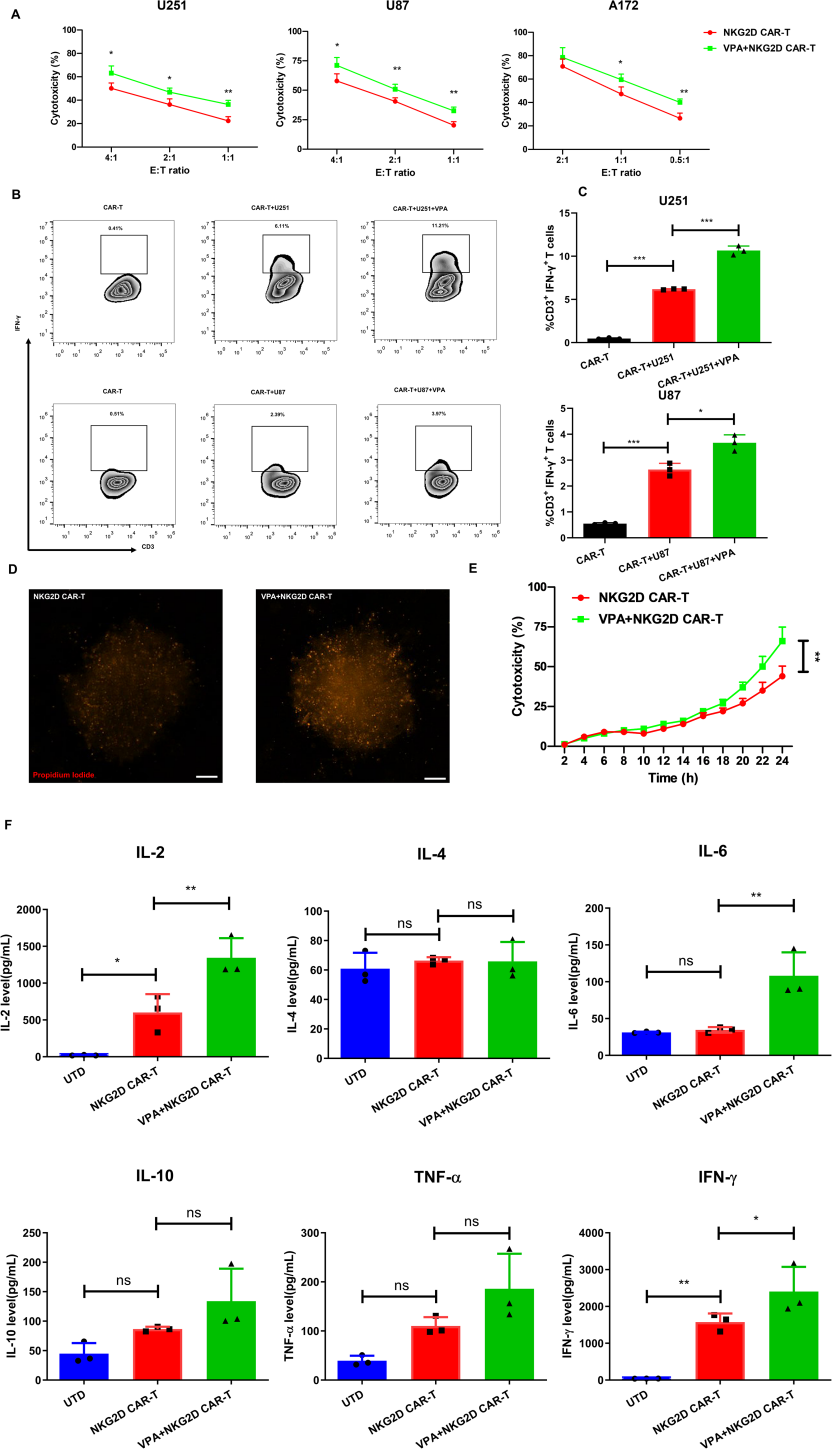
VPA treatment enhances the susceptibility of glioblastoma cancer cells to NKG2D CAR-T cell-mediated attack. **(A)** The cytotoxicity of NKG2D CAR-T cells against the indicated cell lines treated with 3.2 mM VPA or vehicle control for 48 h at different E:T ratios after 24 h co-culture. The results are presented as the mean ± SD (*n* = 3). Statistical significance between the NKG2D CAR-T only group and NKG2D CAR-T plus VPA group was calculated using a two-tailed unpaired Student t test (*P ≤ 0.05, **P ≤ 0.01, ***P ≤ 0.001). **(B, C)** Intracellular FACS analysis of IFN-γ secreting CD3+ T cells after co-culturing CAR-T cells and glioblastoma cell lines for 24 h at an E:T ratio of 1. **(B)** Representative flow cytometry plots of IFN-γ secreting CD3+ T in groups of NKG2D CAR-T only control, NKG2D CAR-T stimulated by cancer cell, NKG2D CAR-T stimulated by cancer cell treated by 3.2 mM VPA for 48 h. **(C)** The summarized statistics data of the frequency of IFN-γ secreting T cells within each population (*n* = 3). Statistical significance was calculated using one-way ANOVA (*P ≤ 0.05, **P ≤ 0.01, ***P ≤ 0.001). **(D, E)** The cytotoxicity of NKG2D CAR-T cells against U251 3D tumor spheroids treated with 3.2 mM VPA or vehicle control for 72 h. The cytotoxicity indicated by fluorescence intensity of propidium iodide was measured by Cytation 5 Cell Imaging Multi-Mode Reader over 24 h co-culture at an E:T ratio of 2. **(D)** Representative images of 3D spheroids stained with propidium iodide after 24 h co-culture with NKG2D CAR-T, scale bar = 100 µm. **(E)** Dynamic time-kill curve of NKG2D CAR-T against 3D tumor spheroids treated with VPA or not. Quantification of cytotoxicity was performed based on the red fluorescent intensity (*n* = 6 spheroids per group). Statistical significance between the NKG2D CAR-T only group and NKG2D CAR-T plus VPA group was calculated using a two-tailed unpaired Student t test (*P ≤ 0.05, **P ≤ 0.01, ***P ≤ 0.001). **(F)** Level of cytokines in the co-culture system of effect and target cells *in vitro* (*n* = 3). Statistical significance was calculated using one-way ANOVA (ns, *P* > 0.05, *P ≤ 0.05, **P ≤ 0.01).

To investigate whether the enhanced expression levels of NKG2DL on the tumor cell surface are associated with their improved recognition and cytotoxicity by NKG2D CAR-T cells, we measured the cytotoxicity of NKG2D CAR-T cells against the tumor cells after co-culture with a subtoxic concentration of VPA or the untreated glioblastoma cancer cells. As expected, VPA-induced NKG2DL upregulation improved antigen-specific recognition and cytotoxicity of NKG2D CAR T cells (**Figure 3A**).

The cytotoxicity of U251 and U87 was significantly increased at all E: T ratios, whereas A172 was significantly increased at E: T ratios of 1 and 0.5 (*P* < 0.05). Additionally, the ability of NKG2D CAR-T cells to produce IFN-γ (assessed via intracellular cytokine staining) was used to assess their activation after co-culture with VPA-treated or untreated U251 and U87 cells. The results indicated that VPA-treated glioblastoma cancer cells can stimulate CAR-T to secrete a higher IFN-γ response compared to the untreated groups (**Figures 3B, C**), further confirming that VPA pretreatment can enhance glioblastoma cell recognition to NKG2D CAR-T mediated immune response.

Furthermore, 3D tumor spheroids have been shown to mimic *in vivo* solid tumor biology more accurately. Spheroids have been shown in studies to be more resistant to chemotherapy than 2D monolayer cell cultures. 3D spheroids develop hypoxic cores and have a drug diffusion profile similar to tumors (19). Based on the above premise, we created a 3D tumor spheroids model with U251 cells and tested the cytotoxicity of NKG2D CAR-T cells after VPA-treated or untreated 3D tumor spheroids. Consistent with the results of the 2D cell model experiments, the 3D tumor spheroids treated with VPA for 48 h were more susceptible to NKG2D CAR-T cells than untreated spheroids (**Figures 3D–F**). Collectively, these findings suggest that VPA treatment increased the susceptibility of glioblastoma cancer cell lines to the antitumor immune response elicited by NKG2D CAR-T cells.

### 3.3 VPA increases the expression of NKG2D ligands in glioblastoma cells via the PI3K/Akt signaling pathway

The expression of NKG2D ligands is linked to various signaling pathways in different cells, including the PI3K/Akt, ERK, HER2/HER3, and P53 pathways (20). VPA has been shown to increase the expression of NKG2D ligands in myeloma cells via an ERK-dependent mechanism (21) and to increase the expression of MICA/B in pancreatic cancer cells via a PI3K/Akt signal pathway (22).

To determine the specific signaling pathway involved in VPA-induced upregulation of NKG2D ligand in glioblastoma cancer cells, we exposed U251, U87, and A172 cells to 3.2 mM VPA for 48 h in the presence or absence of 10 μM of the PI3K inhibitor LY294002, or 20 μM of the ERK inhibitor PD98059, followed by flow cytometry analysis.

**Figure 4:**
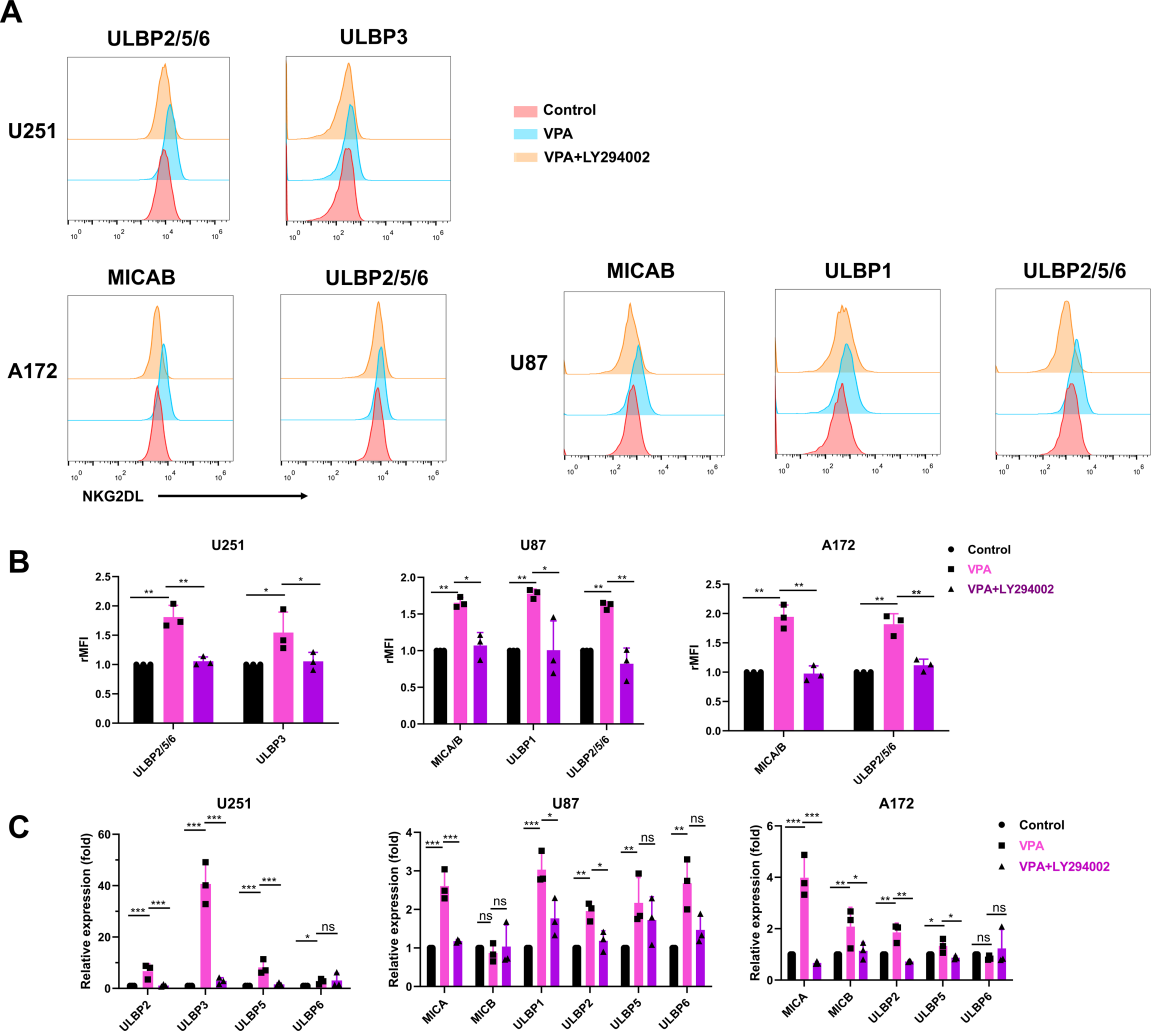
PI3K/Akt signaling pathway is required for VPA induced upregulation of NKG2D ligands expression of glioblastoma cells. Glioblastoma cells were incubated with vehicle, 3.2 mM VPA or 3.2 mM VPA plus 10 μM of the PI3K inhibitor LY294002 for 48 h. **(A, B)** Flow cytometry analysis and quantification of surface NKG2D ligands expression in glioblastoma cells (rMFI, relative mean fluorescence intensity). The results are presented as the mean ± SD (*n* = 3). Statistical significance was calculated using one-way ANOVA (ns, *P* > 0.05, *P ≤ 0.05, **P ≤ 0.01, ***P ≤ 0.001). **(C)** Quantitative real-time RT-PCR analysis of NKG2D ligands expression. The results are presented as the mean ± SD (*n* = 3). Statistical significance was calculated using one-way ANOVA (ns, *P* > 0.05, *P ≤ 0.05, **P ≤ 0.01, ***P ≤ 0.001).

Flow cytometric data showed that LY294002 significantly inhibited VPA’s ability to upregulate the expression of NKG2D ligands (*P* < 0.05, **Figures 4A, B**), whereas PD98059 exposure had no effect on NKG2D ligand expression (data not shown). These findings suggest that the PI3K/Akt signaling pathway, but not the ERK signaling pathway, is involved in VPA-induced upregulation of MICA/B and ULBPs in glioblastoma cancer cells. To support this observation, RT-qPCR analysis revealed that LY294002 significantly reduced the expression of NKG2D ligands upregulated by VPA treatment at the mRNA level (*P* < 0.05, **Figure 4C**). Overall, the data show that VPA probably increases NKG2D ligands expression in glioblastoma cells via the PI3K/Akt signaling pathway.

### 3.4 VPA enhances anti-tumor activity of NKG2D CAR-T cells *in vivo*

**Figure 5:**
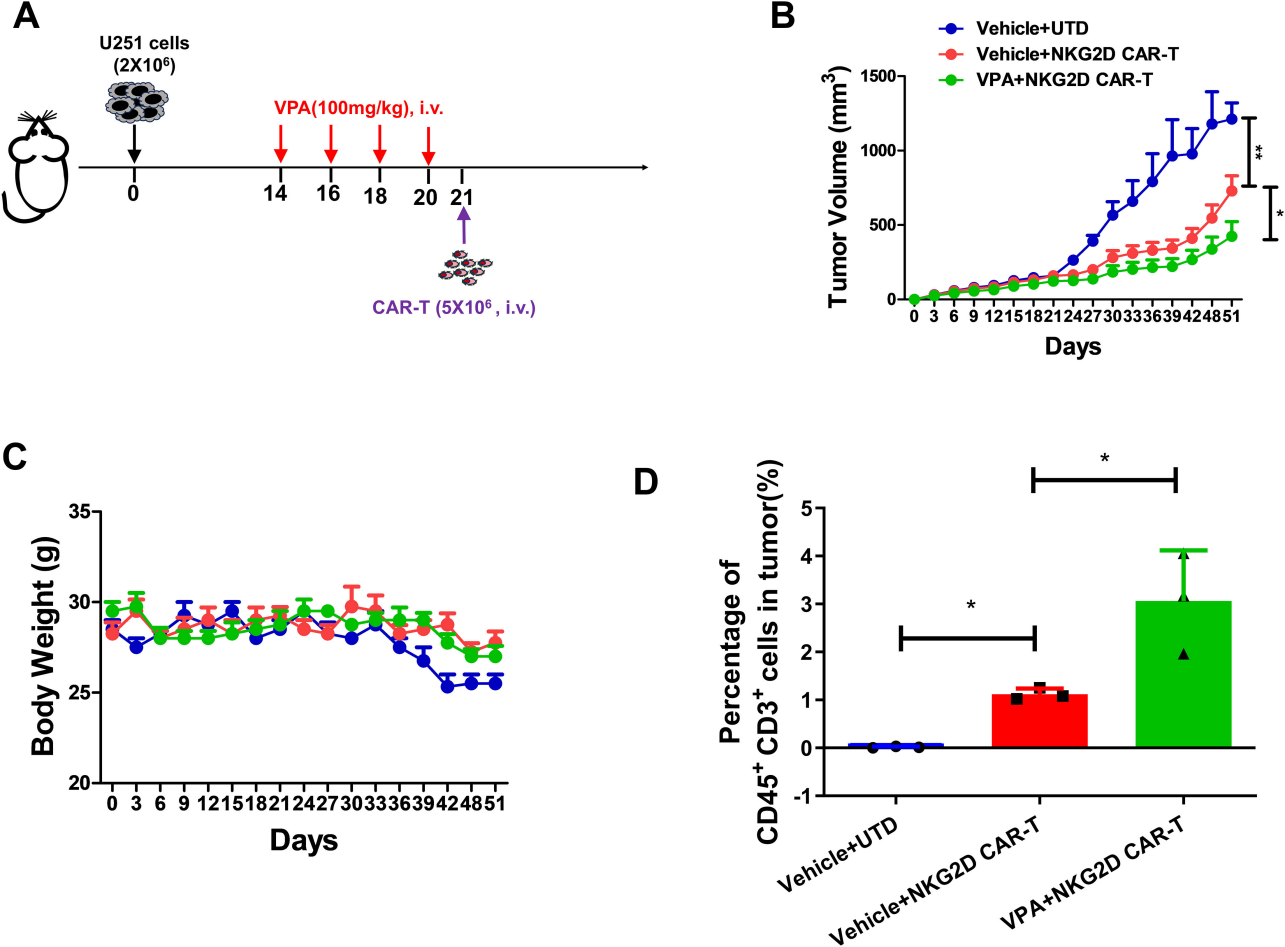
VPA pretreatment enhances the anti-tumor effect of NKG2D CAR-T cells against glioblastoma *in vivo*. **(A)** Schematics of the U251 glioblastoma xenograft model for combinational therapy. B-NDG mice were injected with 2×10^6^ U251 cells via the subcutaneous injection. Then B-NDG mice were treated with VPA via the tail vein, 100 mg/kg or saline every two days for 4 times. Then the mice were infused with 5×10^6^ NKG2D CAR-T cells and UTD cells (*n* = 4). **(B)** Growth curves for the xenograft models (*n* = 4). **(C)** Body weights for the xenograft models. **(D)** Percentage of human CD45+ CD3+ cells in tumor tissues from different groups (*n* = 3). Statistical significance was calculated using one way ANOVA (ns, *P* > 0.05, *P ≤ 0.05).

To assess the anti-tumor efficacy of NKG2D CAR-T in combination with VPA against glioblastoma *in vivo*, a xenograft mouse model bearing U251 cells was established. To determine the dosage of VPA for the animal experiment, a dosage of 100mg/kg were administered intravenously to the mice model every two days, based on the published literature (23). The results showed that tumor growth was not inhibited after 4 times of VPA treatment (**Supplementary Figures S1A, B**). Therefore, the same treatment regime of VPA was used in the following combinational therapy with NKG2D CAR-T cells *in vivo* (**Figure 5A**).

The results revealed that NKG2D CAR-T treatment significantly inhibit tumor growth in B-NDG mice as indicated by reduced tumor volume and tumor weight; and notably, pretreatment of mice with VPA further showed significant tumor growth inhibition (**Figure 5B**; **Supplementary Figures S1C, D**). In addition, no obvious difference in body weight across the different treatment groups was observed (**Figure 5C**), indicating the safety of the combinational therapy. Moreover, analysis of harvested tumor tissues showed that combination of VPA and NKG2D CAR-T treatment remarkably enhanced T cells infiltration into tumor sites as shown by increased CD3^+^ T cells (*P* < 0.005) in tumor tissues (**Figure 5D**; **Supplementary Figure S1E**). Collectively, our findings suggest that VPA potentiates the efficacy of NKG2D CAR-T cells against glioblastoma.

## 4 Discussion

CAR-T immunotherapy has shown promising clinical results in hematologic malignancies. As a result, numerous studies have been conducted to extrapolate the success to solid tumors, including glioblastoma. Solid tumors are expected to have a high level of antigen heterogeneity, and there is usually a risk of the target antigen being destroyed and removed from the cancer cells (24). To date, CAR-T cells targeting EGFRVIII, IL13Ra2 and HER2 have been applied in clinical studies of glioblastoma and have shown promising results in a few patients (5). This demonstrated the feasibility and safety of CAR-T cell-based immunotherapy in glioblastoma treatment. However, these responses have not been consistent, in large part due to heterogeneous tumor antigen expression and escape following treatment with CAR-T cells directed at a single target (25).

Our findings, as well as those of previous studies, show that gliomas cells can overexpress NKG2D ligands that are absent in normal tissues (10). The ability of NKG2D-based CAR-T cells to target multiple tumor antigens expressed in tumor cells (i.e., eight NKG2D ligands) is extremely beneficial in the treatment of glioblastoma. Additionally, NKG2D CAR-T can kill immunosuppressive myeloid-derived suppressor cells and regulatory T cells, as well as tumor neovasculature expressing NKG2D ligands, thereby overcoming the immunosuppressive tumor microenvironment (TME) and increasing immunotherapy efficacy (26). In this study, we first confirmed the overexpression of NKG2D ligands on glioblastoma cells and then constructed a classic second-generation NKG2D CAR using the extracellular domain NKG2D, followed by 4-1BB and CD3ζ.

The generated NKG2D CAR-T demonstrated potent cytotoxicity against glioblastoma cell lines *in vitro*, which is consistent with a recent report that NKG2D CAR-T is effective against glioblastoma (10). However, glioblastoma normally evades NKG2D-mediated immune detection by downregulating or shedding soluble NKG2D ligands from the cell surface (11, 27). In clinical trials of various solid tumors, CAR-T therapy often fails to achieve satisfactory results because of immune escape. CAR-T cell therapy combined with traditional chemotherapy are expected to overcome immunosuppressive TME and immune escape to achieve satisfactory efficacy (28, 29). Combining NKG2D CAR-T cells with therapeutic agents that can upregulate NKG2D ligand expression in glioblastoma cells may overcome tumor antigen heterogeneity, immune escape problems, and significantly improve the efficacy of NKG2DL-directed immunotherapy.

Several small molecule drugs, including VPA, gemcitabine, superpolyamide hydroxamic acid (SAHA), and temozolomide, have been shown to improve NK cell-mediated cytotoxicity against cancer cells by upregulating NKG2D ligands (13, 17). VPA is of particular interest because it is a well-established and well-tolerated drug for long-term epilepsy therapy, including epileptic seizures in glioblastoma patients. In addition, VPA can cross the blood-brain barrier and reach the brain (30). Finally, because seizure is one of the neurotoxic effects of CAR-T therapy (31, 32), VPA has potential to prevent and treat seizure when used in conjunction with CAR-T therapy.

Therefore, we investigated the effect of VPA on the expression of surface NKG2D ligands on glioma cell lines and sought to combine VPA and NKG2D CAR-T to treat glioblastoma. Our findings suggest that VPA can increase the expression of NKG2D ligands in glioblastoma cells at the protein and mRNA levels, making them more susceptible to NKG2D CAR T cell-mediated immune attack *in vitro* and *in vivo*. Noteworthy, the severe immunosuppressive effect in the TME is one of the characteristic features of gliomas, which plays a particularly important role in facilitating evasion of the tumor from the immune response (33). VPA has been shown to effectively relieve the immunosuppressive TME by reducing tumor infiltration of MDSCs and increasing T cell recruitment and activation (34, 35). In our *in vivo* study, CAR-T cell infiltration to the tumor was enhanced by VPA as well. The described combination strategy should be considered as a potential immunotherapy approach to treat glioblastoma, as this study paved the way for further development of similar promising therapeutic approaches.

Three glioblastoma cell lines (U251, U87, A172) were used for *in vitro* studies, while only U251 cells were used for *in vivo* studies in this study. Since glioblastoma is a highly heterogeneous disease, these cell lines may not represent the full spectrum of glioblastoma subtypes. Therefore, more glioblastoma cell lines and primary glioblastoma cells need to be tested further for this combinational therapeutic approach. In our *in vivo* study, a fixed dose of VPA was used based on prior literature. To identify the optimal VPA concentration for enhancing NKG2D CAR-T efficacy *in vivo*, a dose-response analysis could be performed. Although VPA and NKG2D CAR-T have demonstrated good safety in the clinic respectively, a more detailed analysis of potential toxicity for the combinational therapeutic approach needs to be explored comprehensively in the future, for instance, organ-specific toxicity, long-term adverse effects, and histopathological examination of treated animals.

A major concern is the tumor selectivity of antigen expression, which must be maintained after systemic administration of upregulating agents to avoid off-target toxicity (17). It is critical to determine whether VPA treatment induces the expression of NKG2D ligands on normal cells. Previous studies have shown that VPA induces the expression of NKG2D ligands in cancer cells but not in normal cells (13, 36). Nevertheless, the mechanism of preferential VPA-induced upregulation of NKG2DL expression by malignant cells is not well understood. We assume that this effect is due to its ability to affect several cellular processes that are dysregulated in malignant but not in normal cells. In addition, intracranial injection of NKG2D CAR T cells may resolve the safety concerns and reduce the possibility of “on target, off organ” toxicity caused by low-level NKG2D ligands expression on normal tissues.

In our study, we investigated the possible mechanism by which VPA increases the surface expression of NKG2D ligands on glioblastoma cells. We observed that VPA increases the expression of NKG2D ligands in glioblastoma cells, possibly by activating the PI3K/Akt signaling pathway rather than the ERK pathway. However, as VPA is a histone deacetylase (HDAC) inhibitor which may have broad effects on gene expression, other possible mechanisms need to be further explored in the future. Using VPA as a starting point, the mechanism of action study lays the foundation for developing and identifying more potent, selective and safer NKG2D ligands inducers.

Interestingly, several drugs that inhibit the PI3K/Akt signaling pathway have been developed to treat human cancer. Our findings suggest that inhibiting the PI3K/Akt signaling pathway reduces the expression of NKG2D ligands or, at the very least, inhibits the upregulation caused by VPA in glioblastoma cells. Therefore, we advise against using PI3K inhibitors as anti-cancer drugs in patients with glioblastoma who are receiving NKG2DL-mediated immunotherapy, such as NKG2D CAR-T therapy.

In conclusion, this study demonstrated that VPA treatment increased NKG2D ligands expression via the PI3K/Akt signaling pathway in glioblastoma cells, making glioblastoma cells more susceptible to NKG2D CAR-T cell-mediated cytotoxicity *in vitro* and *in vivo*. These findings suggest that combining NKG2D CAR-T cells with VPA could be a viable and promising therapeutic strategy for glioblastoma. Our research provides proof-of-concept data for the use of a novel combination therapy against glioblastoma, which can be explored further in the clinic.

## Data Availability

The original contributions presented in the study are included in the article/**Supplementary Material**. Further inquiries can be directed to the corresponding authors.
