# Supplementary material for: Sodium valproate enhances efficacy of NKG2D CAR-T cells against glioblastoma
Source: Front Immunol. 2025 Jan 14;15:1519777. doi: 10.3389/fimmu.2024.1519777 (PMC11772361; doi:10.3389/fimmu.2024.1519777)
Supplement: Supplementary file 1 [file Table1.docx]

Sodium Valproate Enhances Efficacy of NKG2D CAR-T Cells against Glioblastoma

Supplementary Material

**Table 1.** Primers for quantitative RT-PCR.

| **Genes** | **Forward (5′ -3′ )** | **Reverse (5′ -3′ )** |
| --- | --- | --- |
| GAPDH | GAGTCAACGGATTTGGTCGT | GATCTCGCTCCTGGAAGATG |
| MICA | ATCTTCCCTTTTGCACCTCC | AACCCTGACTGCACAGATCC |
| MICB | CTGCTGTTTCTGGCCGTC | ACAGATCCATCCTGGGACAG |
| ULBP1 | ACTGTGTTAACCACAAGGCCA | CAGGGTGAGGGGCTCAATG |
| ULBP2 | TCATCTTCCAGGCTCTCCTTC | AAAGAGAGTGAGGGTCGGC |
| ULBP3 | AGGAAGAAGAGGCTGGAACC | CTATGGCTTTGGGTTGAGCTA |
| ULBP4 | CAGAACCGACAGTGTCACCA | TGCCACCAGACACAGATGAG |
| ULBP5 | ATCCAACTCCCCAATGGCAG | CAAAGAGAGTGAGGGTCGGC |
| ULBP6 | ACATCACCGTCATCCCTAAGT | GTCTTGTTGCCACAGTCATAGT |


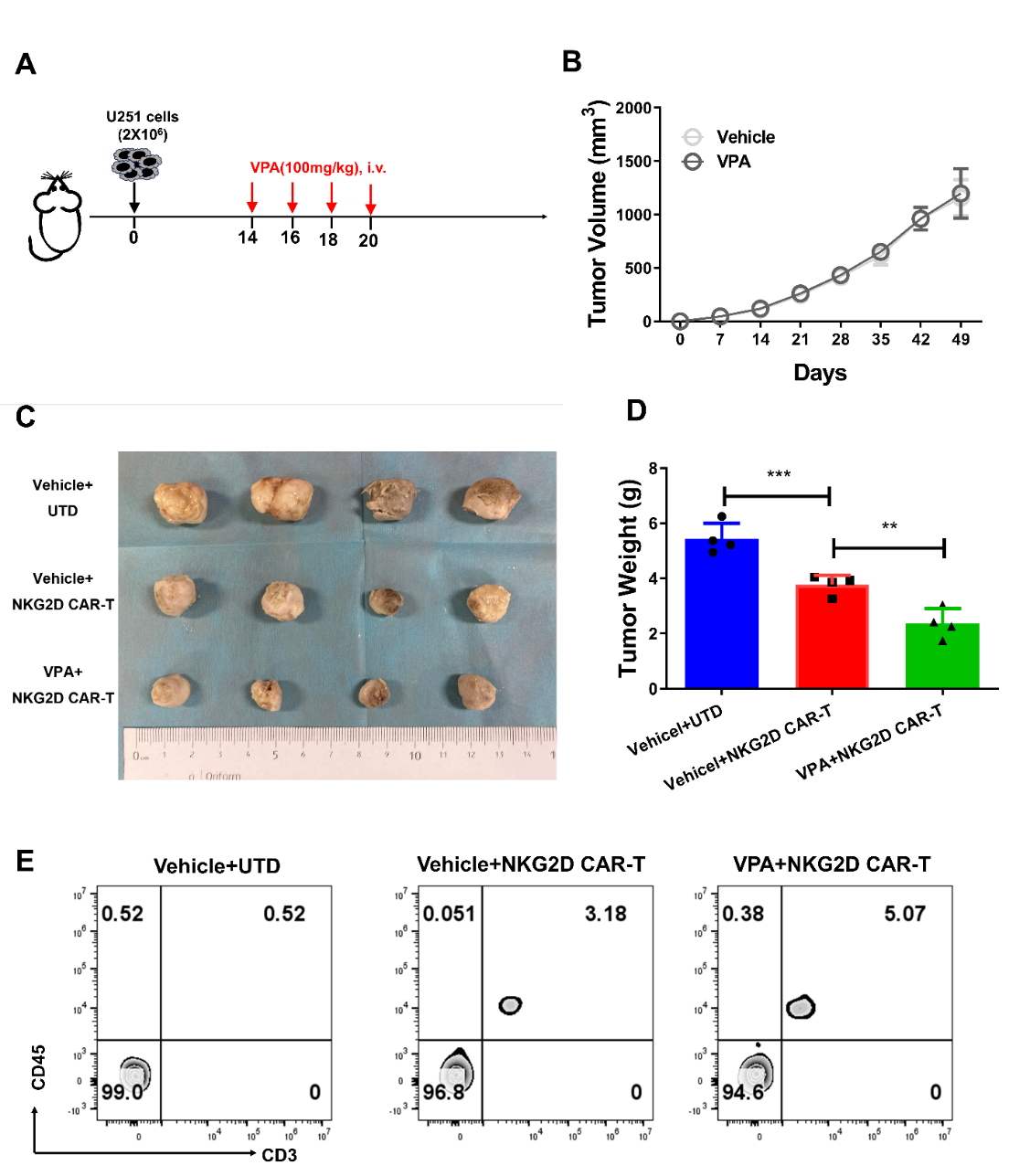


**Figure S1.** **The combination of VPA and NKG2D CAR-T cells for the treatment of glioblastoma in vivo. (A)** Schematics of the U251 glioblastoma xenograft model for VPA monotherapy. B-NDG mice were injected with 2×10^6^ U251 cells via the subcutaneous injection. Then B-NDG mice were treated with VPA via the tail vein, 100 mg/kg or saline every two days for 4 times (*n* = 3). **(B)** Effect VPA treatment on the tumor growth of U251 xenograft models. **(C)** Image of the xenograft tumors from different groups. **(D)** Tumor weight of the xenograft tumors from different groups (*n* = 4). Statistical significance was calculated using one way ANOVA (ns, *P* > 0.05, **P* ≤ 0.05, ***P* ≤ 0.01, ****P* ≤ 0.001). **(E)** Representative flow cytometry plots of the percentage of human CD45^+^ CD3^+^ T in tumor tissues form different groups**.**
